# Feature of Adhesins Produced by Human Clinical Isolates of *Mycobacterium intracellulare*, *Mycobacterium intracellulare* subsp. *chimaera* and Closely Related Species

**DOI:** 10.3390/microorganisms8081154

**Published:** 2020-07-30

**Authors:** Louise H. Lefrancois, Thierry Cochard, Maxime Branger, Olivia Peuchant, Cyril Conde, Adeline Pastuszka, Camille Locht, Philippe Lanotte, Franck Biet

**Affiliations:** 1 INRAE, Université de Tours, ISP, F-37390 Nouzilly, France; louise.lefrancois.phd@gmail.com (L.H.L.); thierry.cochard@inrae.fr (T.C.); maxime.branger@inrae.fr (M.B.); cyril.conde@inrae.fr (C.C.); adeline.pastuszka@univ-tours.fr (A.P.); philippe.lanotte@univ-tours.fr (P.L.); 2 INRAE, Université de Bordeaux, USC EA 3671 Mycoplasmal and Chlamydial Infections in Humans, Laboratoire de Bactériologie, Centre Hospitalier Universitaire de Bordeaux, F-33000 Bordeaux, France; olivia.peuchant@chu-bordeaux.fr; 3 Service Bactériologie-Virologie, Hôpital Bretonneau-CHRU de Tours, F-37044 Tours, France; 4 Univ. Lille, CNRS, Inserm, CHU Lille, Institut Pasteur de Lille, U1019—UMR9017—CIIL—Center for Infection and Immunity of Lille, F-59000 Lille, France; camille.locht@pasteur-lille.fr

**Keywords:** *Mycobacterium intracellulare*, NTM, adhesins, HBHA, LBP, MAC infection

## Abstract

The *Mycobacterium avium* complex includes two closely related species, *Mycobacterium avium* and *Mycobacterium intracellulare*. They are opportunistic pathogens in humans and responsible for severe disease in a wide variety of animals. Yet, little is known about factors involved in their pathogenicity. Here, we identified, purified and characterized adhesins belonging to the heparin-binding hemagglutinin (HBHA) and laminin-binding protein (LBP) family from *M. intracellulare* ATCC13950 and examined clinical isolates from patients with different pathologies associated with *M. intracellulare* infection for the presence and conservation of HBHA and LBP. Using a recombinant derivative strain of *M. intracellulare* ATCC13950 producing green fluorescent protein and luciferase, we found that the addition of heparin inhibited mycobacterial adherence to A549 cells, whereas the addition of laminin enhanced adherence. Both HBHA and LBP were purified by heparin-Sepharose chromatography and their methylation profiles were determined by mass spectrometry. Patients with *M. intracellulare* infection mounted strong antibody responses to both proteins. By using PCR and immunoblot analyses, we found that both proteins were highly conserved among all 17 examined clinical *M. intracellulare* isolates from patients with diverse disease manifestations, suggesting a conserved role of these adhesins in *M. intracellulare* virulence in humans and their potential use as a diagnostic tool.

## 1. Introduction

Non-tuberculous mycobacteria (NTM) are an increasing cause of opportunistic diseases in humans [1,2]. Among NTM, the *Mycobacterium avium* complex (MAC) represents a group with a specific distribution of species according to continent and countries [3]. In addition to severe infection in immune-deficient subjects, such as AIDS patients, the incidence of MAC infections has also recently increased in patients with chronic pulmonary disease and other underlying conditions [4,5]. Due to modifications in mandatory programs of vaccination with bacillus Calmette-Guérin (BCG) in low-incidence countries, an increase in the frequency of adenitis in children was noticed [6] mostly because of MAC infection. MAC is classically divided into *Mycobacterium avium* and *Mycobacterium intracellulare*. The *M. avium* species includes four closely related subspecies, *M. avium* subsp. *paratuberculosis*, the etiologic agent of Johne’s disease or paratuberculosis in ruminants [7]. *M. avium* subsp. *avium* and *M. avium* subsp. *silvaticum*, responsible for avian tuberculosis and infection in wood pigeons, respectively [8] and *M. avium* subsp. *hominissuis*, which is usually isolated from pigs but can also be implicated in human infections [9]. Some recently discovered species are very close to *M. intracellulare* and are termed *M. intracellulare* subsp. *chimaera intracellulare* complex (MCIC) [10,11]. *M. intracellulare* and *M. intracellulare* subsp. *chimaera* are associated with infections in humans. *M. intracellulare* is mainly implicated in pulmonary infections, and *M. intracellulare* subsp. *chimaera* [12], was recently associated with fatal infections after cardiac surgery [13]. MAC can be identified by using DNA probes, luminescent systems, DNA sequencing of *rpoB*, *hsp65* and the 16S–23S Intergenic region, or identification of specific insertion sequences [10]. GenoType NTM-DR, a new commercial diagnostic assay, allows differentiation between three MAC species, *M. avium*, *M. intracellulare*, *M. intracellulare* subsp. *chimaera*, as well as identification of subspecies within the *Mycobacterium abscessus* complex [14]. Mass spectrometry has also been recently proposed as a useful tool to identify these NTM at the species level [15].

Several studies have shown that pathogenic mycobacteria use the protein or proteoglycan component of the extracellular matrix (ECM) for adherence and invasion of the host [16]. One of the best characterized mycobacterial adhesins is the heparin-binding hemagglutinin (HBHA), initially identified in *Mycobacterium tuberculosis* and *Mycobacterium bovis* bacillus Calmette-Guérin (BCG) [17,18]. However, HBHA-like molecules are also present in many other mycobacteria, both pathogenic and nonpathogenic [19,20,21,22]. HBHA is located on the surface of the mycobacteria and mediates binding of the bacilli to epithelial cells and fibroblasts [18] by interacting with sulfated glycoconjugates present on the surfaces of host cells [23]. It also plays a role in the dissemination of *M. tuberculosis* from the lungs to deeper tissues [24] and has shown promise as a diagnostic target for the detection of latent tuberculosis in humans [25,26,27,28].

Laminin and collagen in the lung also promote adherence to ECM-binding mycobacteria, and mycobacterial laminin-binding proteins (LBP) involved in adherence of mycobacteria to host cells have been identified and characterized. LBP was initially described to play a role in the interaction between *Mycobacterium leprae* and Schwann cells [29,30,31]. LBP, also referenced to as Lbp/Hlp [32,33], Mdp1, the Mycobacterial DNA-binding protein 1 [30] and hupB the mycobacterial histone-like protein are conserved in mycobacteria, including MAC [32].

In this study, we characterized HBHA and LBP from MCIC species isolated from patients with a variety of disease expression, examined the role of these adhesins in the binding of *M. intracellulare* to lung epithelial cells and their degree of conservation within the MCIC.

## 2. Materials and Methods

### 2.1. Bacterial Strains and Growth Conditions

**Figure 1:**
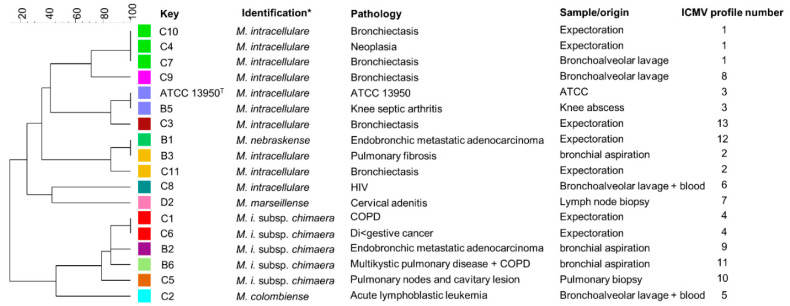
Genetic diversity of clinical strains. Clinical isolates were genotyped by Multilocus Variable numbers tandem repeats Analysis (MLVA) based on 7 markers. The MLVA-based polymorphism dendrogram was made with Bionumerics. MLVA profiles are represented by a color. key, strain name; ICMV, *M. intracellulare* MLVA, * Species were identified by *hsp65* sequencing. T, type strain. Chronic obstructive pulmonary disease (COPD).

The reference strain of *M. intracellulare* ATCC13950 was from the American Type Culture Collection (ATCC), USA. The MAC clinical isolates from AIDS patients and from non-HIV infected patients (n = 17) were collected from inpatients hospitalized at Tours University hospital and Bordeaux University Hospital (France). The protocol was submitted to the local Ethics Committee (protocol authorization no. 2020-062 from the CHU-Tours Ethics Committee in Human Research). No written consent was requested as there was no clinical intervention regarding the healthcare and the study was retrospective. The origin of the samples and clinical context are presented in Figure 1 and Table 1.

*M. intracellulare* ATCC13950 and human clinical isolates were grown at 37 °C in Middlebrook 7H9 broth (Difco Laboratories, Detroit, MI, USA), with 0.2% glycerol and 10% of ADC (albumin–dextrose–catalase enrichment medium, Becton Dickinson, Le Pont-de-Claix, France). Bacteria were harvested at mid-log phase and kept frozen (−80 °C) with 15% glycerol in aliquots until use.

### 2.2. Strain Identification and Genotyping

#### 2.2.1. Species Identification by GenoType CM and Sequence Analysis of the *hsp65* Gene

Identification was performed routinely using GenoType CM (Hain, Life Science). The identification at the species level was performed by *hsp65* sequencing. The method for the rapid identification of MAC species was adapted from Talenti et al. [34]. For the PCR, 100 µL of culture in a stationary phase was frozen at −80 °C for 1 h then bacteria were heat-killed for 15 min at 95 °C. PCRs were performed with 5 μL of DNA from the thermolysate supernatant added to a final volume of 50 μL containing 0.2 μL of GoTaq Flexi DNA polymerase (5 U/μL), 2 µL dimethyl sulfoxide (DMSO), 2 mM of dATP, dCTP, dGTP and dTTP (Promega); 10 μL of 5× PCR buffer supplied by the manufacturer; 1 μM of primers P1 and P2 (Table 2) and 1.5 mM of MgCl2. The reactions were carried out using a TC-512 thermal cycler (Techne). PCR conditions were as follows: 1 cycle of 5 min at 94 °C; 30 cycles of 30 s at 94 °C, 30 s at 58 °C and 30 s at 72 °C; and 1 cycle of 7 min at 72 °C.

PCR products (441 bp) were visualized by electrophoresis using 1.5% agarose gels (agarose electrophoresis grade; Invitrogen), purified using NucleoSpin Extract II (Macherey-Nagel) and sequenced by GenomExpress (Grenoble, France). Sequences were analyzed using the leBIBI-QBPP website https://umr5558-bibiserv.univ-lyon1.fr/lebibi/lebibi.cgi. Phylogeny was performed with a concatenate of SNP using the software Bionumerics® v. 7.6.3 (Applied Maths).

#### 2.2.2. MLVA Genotyping

A Multilocus Variable numbers tandem repeats Analysis (MLVA) profile identification was carried out according to Dauchy et al. [35], using PCR amplification target 7 genetic loci designed from the genome of *M. intracellulare*. The PCR reaction was composed of 1 U GoTaq Flexi DNA polymerase (Promega), 1 μM of each primer (Table 2), 1 μM dNTP, 5 μL 5× buffer solution; 1.5 mM MgCl2, 1 μL DMSO (Sigma); and distilled H_2_O qs 20 µL. The mixture was added to 5 μL of DNA. Amplification conditions were as follows: 1 cycle of 5 min at 94 °C; 30 cycles of 30 s at 94 °C, 30 s at 58 °C and 30 s at 72 °C; and 1 cycle of 7 min at 72 °C. To detect the difference in repeat numbers, the PCR products were analyzed, by electrophoresis in a 1% agarose gel, by the use of Gel Doc (Bio-Rad, Hercules, CA, USA) and Quantity One 4.2.1 (Bio-Rad, Hercules, CA, USA) software for fragment size calculation. Phylogenetic relationships between the different isolates were analyzed using the software Bionumerics® v. 7.6.3 (Applied Maths, Sint-Martens-Latem, Belgium).

### 2.3. PCR DNA Fragment Analysis

To confirm in silico data from available genomes, detection and size analysis of the *hbhA* and *lbp* genes was investigated by PCR as described [20]. The sizes of the PCR products corresponding to *hbhA* and *lbp* were determined after the amplification of genomic DNA of all isolates with primers described in Table 2. The fragments were amplified after a denaturation cycle of 5 min at 94 °C by using only 23 cycles of 94 °C for 30 s, 61 °C for 30 s and 72 °C for 30 s, followed by a final elongation cycle at 72 °C for 5 min. The size of the PCR products was analyzed by electrophoresis using 1.5% agarose gels (agarose electrophoresis grade; Invitrogen, Carlsbad, CA, USA).

### 2.4. Construction of Bioluminescent and Fluorescent M. intracelullare Strain

Green fluorescent protein (GFP)- and luciferase-expressing bacteria were obtained by electroporation of the pSMT3LxEGFP [36] carrying both the GFP- and the bacterial luciferase-coding genes (Lux) into *M. intracelullare* ATCC 13950. Bacterial cultures (150 mL) were grown to mid-log phase in Sauton medium and centrifuged at 4500 × g for 15 min. The pellets were resuspended in 150 mL of medium supplemented with 10% glycerol. Cells were centrifuged again, washed three times with 75 mL of medium supplemented with 10% glycerol, and resuspended in 5 mL 10% glycerol. Electroporation-ready mycobacterial cells were aliquoted and frozen at −80 °C until further use. For transformation, 1 µg of pSMT3LxEGFP DNA was mixed with 50 µL of cell suspension and pulsed with a Cellject electroporator (Eurogentec, Liège, Belgium) at 2.5 kV, 800 Ω and 25 µF. The cells were then harvested in 1.0 mL Sauton medium and plated onto Middlebrook 7H10 medium supplemented with ADC and 50 µg ml^−1^ of hygromycin. The plates were incubated at 37 °C and colonies were isolated after 30 to 40 d.

### 2.5. Cytoadherence of M. intracelullare to A549 Epithelial Cells

A549 cells were grown at 37 °C in an atmosphere of 5% CO_2_, in DMEM (Lonza) containing 10% heated fetal bovine serum (Invitrogen), and supplemented with 2 mM L-glutamine. Twenty-four hours before the adhesion assay, 1.5 × 10^5^ cells/well were seeded in a 24-well plate. The mycobacteria were grown in static conditions in enriched 7H9 Middlebrook broth supplemented with 0.05% Tween 80. At 0.5 of optical density at 600 nm, bacteria were washed once with phosphate-buffered saline (PBS, 10 mM phosphate buffer pH 7.2, 0.15 M NaCl) and resuspended in DMEM. The suspension was vigorously shaken and passed through a 27-G needle three times to disrupt bacterial aggregates. The presence of isolated mycobacterial cells was confirmed by microscopic observation. Confluent monolayers were washed twice by PBS before the addition of the appropriate fluorescent-bioluminescent *M. intracellulare* /GFPlux strain (green). The bacterial concentration was adjusted in DMEM and monolayers were infected at a multiplicity of infection of 100 in presence or absence of 80 µg ml/1 heparin or 20 µg mL/1 laminin. After 3 h of contact at 37 °C in an atmosphere of 5% CO_2_, the infected cells were washed three times with PBS and then lysed for 10 min in 0.1% Triton X-100 followed by another 10 min after addition of an equal volume of 0.025% SDS. The percentage of adhesion was estimated by fluorescence and calculated by luciferase assays, as described below. Cytoadherence of *M. intracellulare* to A549 cells was observed by fluorescence microscopy. The samples were fixed with paraformaldehyde (PFA) and stained with Evans Blue yielding red fluorescence. Images were taken with a 40× objective.

To quantify the cytoadherence of *M. intracellulare* to A549 cells luminescence was measured using a Berthold Lumat LB 9507 luminometer. As needed, the crude cell lysates were diluted in PBS containing 0.05% Tween 80. Aliquots (100 µL) of each sample were transferred into a polystyrene test tube (Falcon, PLAM0908) containing 900 mL dilution buffer and the luciferase activity was measured. Raw data were collected in triplicate and the mean Relative Luminescence Unit (RLU) readings were automatically calculated by the luminometer in-core software. Adhesion assays were performed in triplicate repeated in three independent experiments. The percentage of adhesion was calculated by the following formula: (cell-associated RLU/RLU of the inoculum) × 100. The average and the standard deviation were calculated with the GraphPad Prism software version 6.7.

### 2.6. Purification by Heparin-Sepharose Chromatography

*M. intracellulare* adhesins were purified by heparin-Sepharose chromatography using a frozen pellet of *M. intracellulare* lysate as described previously [37]. All chromatographic steps were carried out on the Biologic chromatography system (Bio-Rad, Hercules, CA, USA) at room temperature, and the absorbance at 260 nm was continuously monitored during purification using the HiTrap Heparin HP (1 mL, GE Healthcare) column (0.7 × 2.5 cm) prepacked with heparin-Sepharose. The column was washed with 100 mL of 20 mM Tris-HCl (pH 7.2) until the absorbance at 280 nm was close to 0. The bound material was eluted by a 0-1M NaCl linear gradient in 20 mM Tris-HCl (pH 7.2), with a flow rate at 0.6 mL min-1 and automatically collected in 1 mL fractions. Whole-cell lysates, flow-through material and eluted fractions were analyzed by sodium dodecyl sulfate-polyacrylamide gel electrophoresis (SDS-PAGE) using 12% polyacrylamide gels.

### 2.7. Identification of the Purified M. intracellulare Proteins by High-Resolution Mass Spectrometry Analysis 

Proteins were digested in-gel with trypsin as previously described [38]. Peptides were analyzed by nanoLC-MS/MS using an Ultimate® 3000 RSLC coupled to a linear ion trap–orbitrap mass spectrometer (LTQ Velos Orbitrap Mass Spectrometer, Thermo Scientific, Waltham, MA, USA). Protein identifications and post-translational modifications were established as previously described [37].

### 2.8. SDS-PAGE and Immunoblotting

To detect the production of HBHA and LBP mycobacterial whole-cell lysates were resolved by SDS-PAGE, using 12% running gels and 4% stacking gels. Proteins were then electro-transferred onto nitrocellulose membranes (Whatman, Germany), followed by a blocking step with 1% BSA in PBS/0.05% Tween, washed three times in PBS/0.05% Tween and then incubated with the monoclonal antibody 3921E4 [39,40]. After three washing steps with PBS/0.05% Tween, membranes were incubated with goat anti-mouse alkaline phosphatase-conjugated antibody (Caltag, Burlingame, CA, USA) at a 1:2000 dilution. The substrates nitro blue tetrazolium and 5-bromo-4-chloro-3-indolyl phosphate were used to develop the immunoblots.

### 2.9. ELISA

ELISA was performed on 96-well Maxisorp microtiter plates (Nunc, Roskilde, Denmark) coated with native HBHA and LBP purified from *M. intracellulare* in at 0.1 mg ml/l in PBS or coated with 50 µL of a purified protein derivative from *M. avium* subsp. *paratuberculosis* (Johnin PPD [PPDj]) (National Veterinary Institute, Oslo, Norway) at 25 mg/L in PBS at 4 °C overnight. Plates were then washed three times with PBS/Tween20 0.05% (PBS/T) and blocked for 1 h at 37 °C with PBS/T containing 0.5% (w/v) gelatin (PBS/T/G). Sera controls were obtained from healthy donors with no context of infection/colonization with MAC. Each serum sample including the sera of the 17 patients infected with MAC, for which the strains were analyzed in this study and sera from 6 healthy controls was diluted at 1:100 in 50 µL PBS/T/G, and plates were incubated for 2.5 h at 37 °C. Plates were then washed five times with PBS/T and incubated for 90 min at 37 °C with 50 mL Horseradish peroxidase-conjugated goat antihuman-IgG, AHu/Ig/PO (Nordic Immunological Laboratories, The Netherlands) diluted at 1:500 dilution in PBS/T/G. Plates were washed five times with PBS/T, and 50 µL 2,2’-Azinobis 3-ethylbenzothiazoline-6-sulfonic acid-diammonium salt (ABTS, Thermo Scientific, Waltham, MA, USA) was added according to supplier’s recommendations. After 15 min incubation at 37 °C, the plates were read photometrically at 414 nm.

### 2.10. Nucleotide Sequence Accession Numbers

The *hbhA* and *lbp* genes were amplified by PCR using primers P19, P22, P23 and P24 (Sigma) (Table 2) and chromosomal DNA of *M. intracellulare* 13950 as a template by using Pfu DNA polymerase (Promega). The sequences of *hbhA* and *lbp* (Genome Express, Takeley, UK). have been deposited in the GenBank databases under the following accession numbers: KY748358 and KY748359.

## 3. Results

### 3.1. Adherence of M. intracellulare to Epithelial Cells is Modulated by Heparin and Laminin

**Figure 2:**
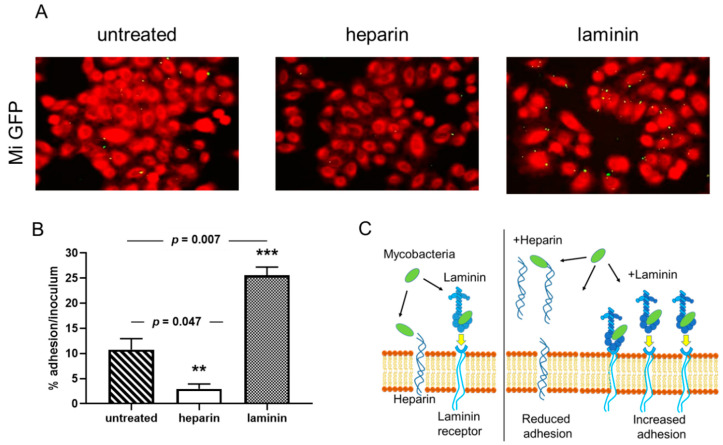
Cytoadherence of *M. intracellulare* ATCC13950 to A549 epithelial cells inhibited heparin and increased by laminin. A549 cells were infected by Green fluorescent protein (GFP)- and luciferase-producing *M. intracellulare* ATCC13950 in the presence or absence of heparin or laminin. (**A**) Fluorescence microscopy analysis of the A549 cells infected by *M. intracellulare* /GFPlux (green). The samples were fixed with PFA and stained with Evans Blue (red). Images taken with 40× objectives represent the overlay of Evans Blue and GFP signals. (**B**) Quantification of *M. intracellulare* /GFPlux adherence by luciferase assays. The percentages of adhesion were calculated by the formula (cell-associated RLU/RLU of the inoculum) × 100. The graph shows the averages of triplicate samples from one representative of three independent experiments. ** *p* < 0.05; *** *p* < 0.01. The error bars represent the standard deviation. (**C**) The diagram gives an illustration of how bacteria bind to cells, either directly on the heparin present on the surface of cells or via laminin which then binds to its cell receptor. In the presence of exogenous heparin or laminin, the adhesion of the bacteria is inhibited or increased.

Previous reports have shown that adherence of mycobacteria to epithelial cells can be modulated by the addition of an extracellular matrix component [18,32]. Using the recombinant *M. intracellulare* strain, we tested whether soluble exogenous heparin or laminin can affect the cytoadherence of *M. intracellulare* to A549 epithelial cells. As observed qualitatively by fluorescence microscopy in Figure 2A, the recombinant *M. intracellulare* (green) is able to adhere to the A549 epithelial cells, stained with Blue Evans (red). Adherence is inhibited by the addition of heparin and enhanced in the presence of laminin. Quantification of *M. intracellulare* adherence by luciferase assay indicated that this adhesion to epithelial cells was significantly decreased from 50 to 60% in the presence of exogenous heparin but increased from 35 to 50% in the presence of exogenous laminin (Figure 2B). The diagram in Figure 2C explains how heparin and laminin can modulate the adhesion of bacteria to cells. The addition of heparin decreases the adhesion of bacteria to cells because it represents targets in competition with the heparin present in cell membranes. Conversely, the addition of laminin will indirectly increase the adhesion of bacteria to the cells via the laminin receptor (Figure 2C).

### 3.2. Purification and Characterization of HBHA and LBP from M. intracellulare by Heparin-Sepharose Chromatography

**Figure 3:**
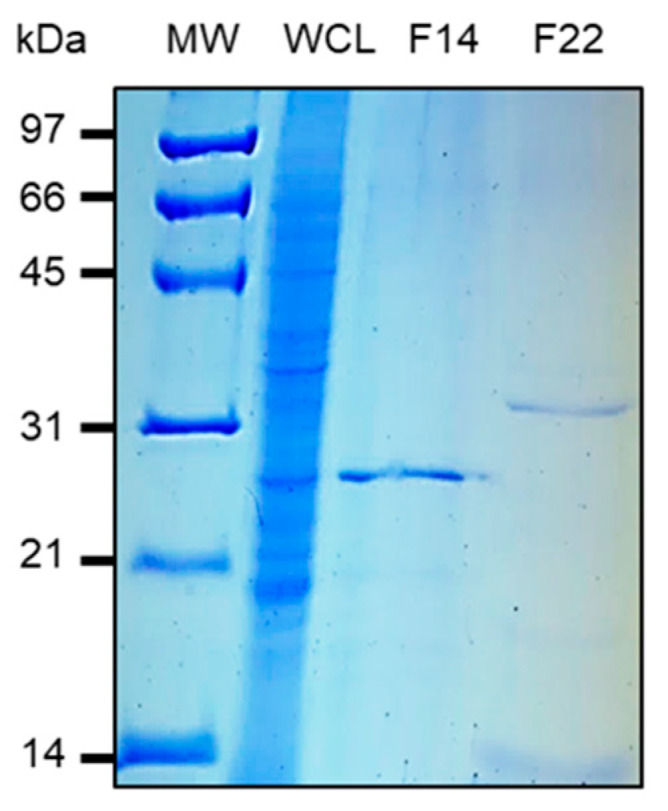
*M. intracellulare* ATCC 13950 heparin-binding hemagglutinin (HBHA) and laminin-binding protein (LBP) purified by heparin-Sepharose chromatography. A 250 mL volume of *M. intracellulare* ATCC 13950 culture was centrifuged and the pellet was resuspended in PBS 0.5×. The bacteria were then sonicated and centrifuged. The whole-cell lysate (WCL) was subjected to heparin-Sepharose HiTrap chromatography. SDS-PAGE analysis shows the eluted material in fraction 14 (F14) at ca. 300 mM NaCl contains *M. intracellulare* HBHA, as determined by HR-MS/MS whereas the *M. intracellulare*-LBP was identified in fraction 22 (F22), eluted at ca. 600 mM NaCl. The molecular weights (MW) expressed in kDa are indicated in the left margin.

The modulation of adherence of *M. intracellulare* to epithelial cells by heparin and laminin suggests that *M. intracellulare* expresses adhesins on its surface, which could be homologs of HBHA and LBP. Thus, to identify and isolate the *M. intracellulare* protein leading to adherence modulation, we performed heparin-Sepharose chromatography on a soluble extract of *M. intracellulare*, as described to purify HBHA and LBP from other mycobacteria [32,37]. As shown in Figure 3, SDS-PAGE analysis on elution fractions 12 to 18 at ca. 300 mM NaCl and in fraction 20 to 24 at ca. 600 mM NaCl contain proteins identified by Mass spectrometry analysis as HBHA and LBP, respectively. Mass spectrometry analysis also confirms the presence of post-translational modifications carried by the HBHA and LBP produced by *M. intracellulare.* As indicated in Table 3 mass spectrometry analysis revealed methylated peptides within the C-terminal lysine-rich repeats domain of HBHA and LBP.

### 3.3. Conservation of HBHA and LBP within the Panel of Clinical M. intracellulare Isolates and Closely Related Species

**Figure 4:**
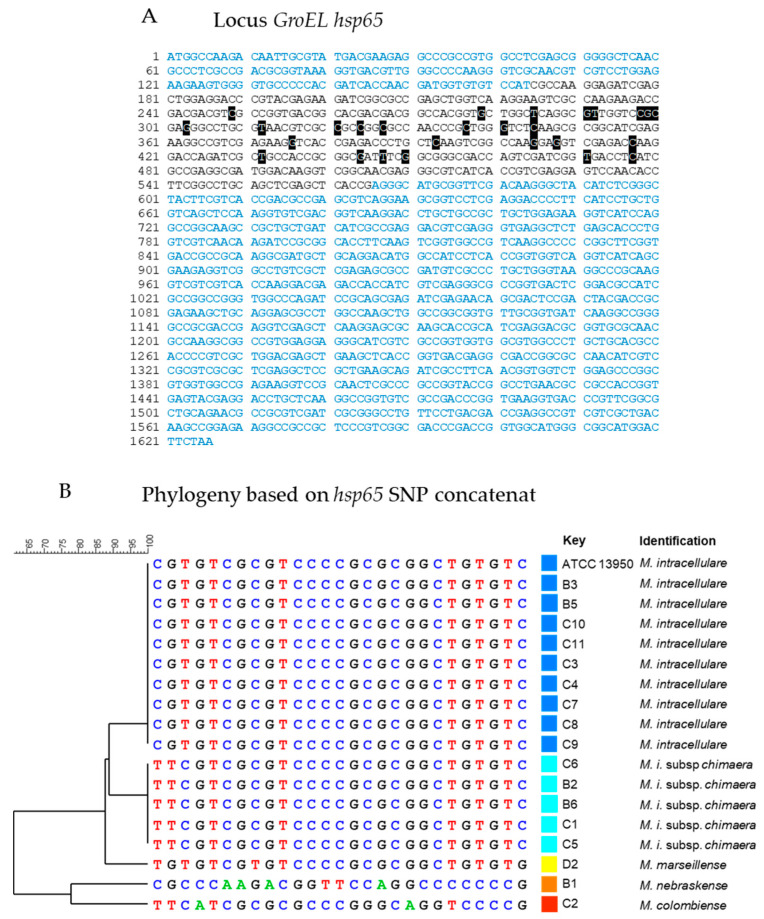
Identification of clinical isolates at the species level by *hsp65* sequence analysis. (**A**) The *hsp65* sequences are indicated in black characters in the *groEL* locus (blue characters). All SNPs detected in *hsp65* of the 17 clinical isolates are indicated by white letters on the black background. (**B**) Alignment of the SNP concatenates detected in *hsp65* associated with the mycobacterial species identified in the BIBI-QBPP database and SNP-based phylogeny carried out with the Bionumeric software.

All clinical isolates used in this study were identified by GenoType Mycobacterium CM (HAin Lifescience) as *M. intracellulare*, but we observed high genetic diversity of clinical isolates when subjected to MLVA genotyping (Figure 1). Using SNP analyses on the *hsp65* gene sequence we found that nine isolates belong to the species *intracellulare.* However, five isolates were reclassified to the *M. intracellulare* subsps. *chimaera*, one to species *marseillense*, one to species *colombiense* and one to species *nebraskense* (Figure 4).

**Figure 5:**
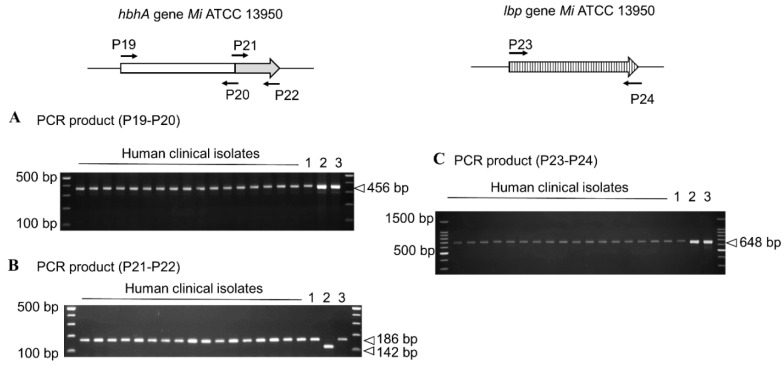
Conservation of the *hbhA* and *lbp* genes in human clinical isolates. PCR amplicons of the DNA coding for the N-terminal (**A**) and the C-terminal domains (**B**) of *hbhA*, and (**C**) of *lbp* obtained by using genomic DNA of *M. intracellulare* ATCC 13950 and lysates of 17 human isolates, as well as genomic DNA from *M. avium* subsp. *paratuberculosis* and *M. avium* subsp. *avium* indicated by 1,2 and 3, respectively. The arrows in the diagram at the top indicate the positions and directions of primers. Positions on a 100-bp ladder (Promega) (**A**,**C**) and a low-molecular-weight DNA ladder (New England BioLabs) (**B**), are indicated in the left margins. The sizes of the PCR products are indicated in the right margins.

Previous studies on HBHA and LBP have shown that within the MAC complex the structure and function of these adhesins can vary according to the species and even within the same subspecies, as in *M. avium* subsp. *paratuberculosis* [20]. This variability was mostly carried by the C-terminal parts of HBHA and LBP, with a different number of lysine residues that lead to variable C-terminal domain lengths and directly impact the binding affinity to heparin [20,23]. We thus investigated by PCR the potential of polymorphisms in the *hbhA* and *lbp* genes of these two adhesins. As Figure 5 illustrates, the sizes of the 3′ region of the *hbhA* and *lbp* genes, corresponding to the portion encoding the heparin-binding protein domains, exhibited the same size for all clinical isolates.

**Figure 6:**
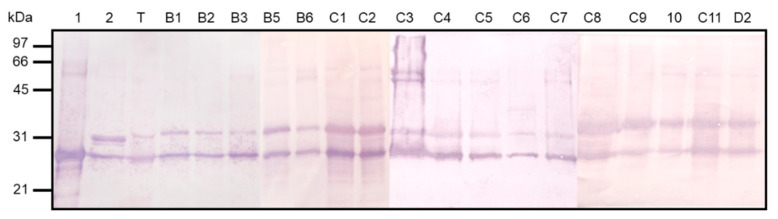
Detection of HBHA and LBP from clinical isolates by immunoblot analysis. HBHA and LBP purified from *M. intracellulare* ATCC13950 (Lanes 1 and 2, respectively); whole-cell lysate of type strain *M intracellulare* ATCC13950 used as control (lane T); whole-cell lysates of human clinical isolates (lanes B1 to D2). The immunoblots were probed with monoclonal antibody 3921E4. Sizes of molecular weight markers (kDa) are indicated in the left margin.

To investigate the expression of *hbhA* adhesin and *lbp* for each clinical isolate, we used a monoclonal antibody able to recognize both HBHA and LBP in immunoblot analyses and found that all the isolates produced the two adhesins at a comparable level and of with the same apparent size (Figure 6).

### 3.4. Both HBHA and LBP are Recognized by Sera from Infected Patients

**Figure 7:**
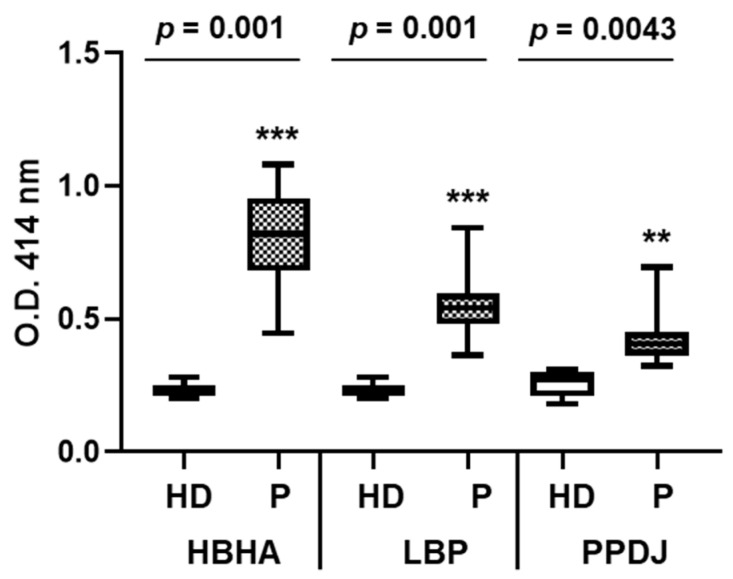
Human serum antibody levels to HBHA and LBP from *M. intracellulare*. ELISA were performed on plates coated with PPDj, HBHA or LBP purified from *M. intracellulare*. The panel of sera tested included 6 healthy donors (HD) and the 17 patients included in this study (P). Serum samples were realized in triplicate. Statistical analysis was performed with Tukey’s multiple comparison tests. ** *p* < 0.005, *** *p* = 0.001.

To investigate whether HBHA and LBP produced by *M. intracellulare* are antigenic in infected subjects, we performed ELISA using sera from 17 infected patients and six healthy controls on *M. intracellulare* HBHA and LBP and PPDj. The sera from the patients reacted with both HBHA and LBP purified from *M. intracellulare* (Figure 7), and the absorbance for the patients’ group was significantly higher compared to that of the healthy donors.

## 4. Discussion

**Figure 8:**
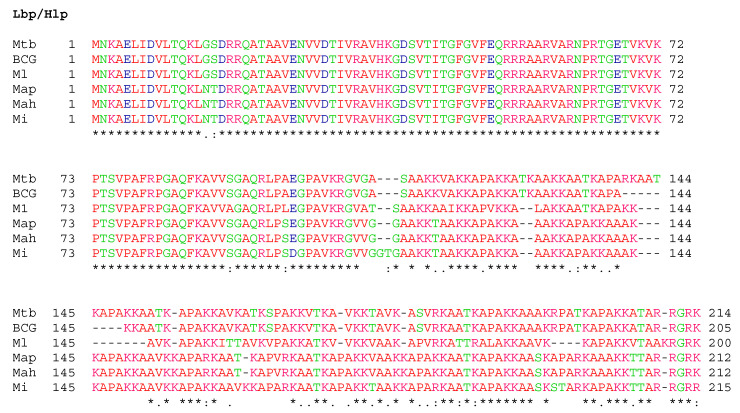

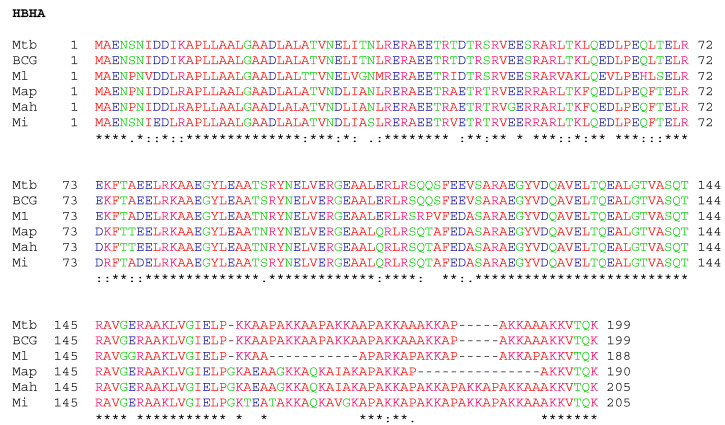
Sequence alignments of HBHA and LBP. Multiple sequence alignments were performed using the ClustalW program with the BLOSUM64 matrix allowing gaps (−). * indicate identical residues.: indicate conserved substitutions.; indicate semi-conserved substitutions. Mtb, *M. tuberculosis*; BCG, *M. bovis* BCG; Ml, *M. leprae*; Map, *M. avium* subsp. *paratuberculosis*; Mah, *M. avium* subsp. *hominissuis* and Mi, *M. intracellulare*. Color code: Pink, Polar positively charged residues HKR; Green, Polar non-charged residues GNQSTY; Blue, Polar negatively charged residues DE and Red, other residues.

One of the major outcomes of this study is the first characterization of HBHA and LBP of *M. intracellulare*. Interaction of *M. intracellulare* with airway epithelial cells can be modulated by heparin or laminin. The addition of exogenous heparin competed with the heparan sulfates present on the proteoglycan components of the cell surface, thereby reducing binding of the bacteria on epithelial cells. Conversely, adding laminin attaches to the bacteria and then binds to its cellular receptor, thereby increasing the adhesion of the bacteria to epithelial cells. Mass spectrometry analysis of heparin-Sepharose-purified *M. intracellulare* HBHA and LBP revealed the characteristic features of these conserved mycobacterial proteins. The affinity properties of these adhesins to sulfated heparin are comparable to their homologs described in *Mycobacterium tuberculosis* Complex (MTBC) and *Mycobacterium leprae* [19,23,41]. These adhesins possess a typical C-terminal domain consisting essentially of lysine/ alanine/proline repeats involved in the interaction with GAGs [23] (Figure 8). Pethe et al. [41], Lebrun et al. [23] and Lefrancois et al. [20] have shown a direct correlation between the number of lysine-rich repeats and the strength of heparin-binding of HBHA. Interestingly, the binding of LBP to laminin and collagen I suggests that this protein may also interact with the host basal membrane. This has been also described for the *Borrelia burgdorferi* protein CRASP-1 which favors adherence and tissue invasion via interaction with laminin and collagen I [42].

HBHA and LBP purified from *M. intracellulare* were found to be methylated, similar to the *M. tuberculosis* homologs [32,43]. The methylation of the functionally important domain of HBHA has been shown to be crucial for protection against proteolytic degradation [43]. In addition, the methylation of HBHA was shown to play a major role in both B and T cell antigenicity [26]. B cell responses were detected here in all patients infected with MAC strains but without differences regarding the pathology. This is not surprising given the high level of degree of similarity of the protein within the different species (Figure 8). Previous studies have shown that native, methylated HBHA from BCG can detect individuals latently infected by *M. tuberculosis* [25]. In fact, the C-terminal, methylated peptide epitopes of the *M. tuberculosis* HBHA are directly recognized by T-cells from latently infected subjects [44]. Furthermore, native methylated *M. tuberculosis* HBHA has been shown to provide protective immunity against *M. tuberculosis* infection in mouse models [45]. Thus, the similarities of the *M. intracellulare* HBHA with the *M. tuberculosis* HBHA suggest the potential usefulness of the latter for diagnosis and vaccine development against NTM infection.

This study also revealed that HBHA and LBP do not differ among the different species belonging to the MAC complex, even though the strains were isolated from patients with different pathologies such as AIDS, bronchiectasis, leukemia and immunosuppressive pathology among others.

## 5. Conclusions

This study describes for the first time the characterization of the adhesins HBHA and LBP of *M. intracellulare* and closely NTM strains. Due to the high degree of conservation, the *hbhA* and *lbp* genes could be targets for the detection of these bacteria in clinical samples, and the corresponding proteins could be targets in therapeutic and vaccine purposes.

## Figures and Tables

**Table 1:** Bacterial strains and plasmid.

Strains	Identification	Pathology	Specimen/Sample	Reference
B1	*M. nebraskense*	Endobronchic metastatic adenocarcinoma	Expectoration	This study
B2	*M. i.* subsps *chimaera*	Endobronchic metastatic adenocarcinoma	bronchial aspiration	This study
B3	*M. intracellulare*	Pulmonary fibrosis	bronchial aspiration	This study
B5	*M. intracellulare*	Knee septic arthritis	Knee abscess	This study
B6	*M. i.* subsps *chimaera*	Multikysitic pulmonary disease + COPD *	bronchial aspiration	This study
C1	*M. i.* subsps *chimaera*	COPD	Expectoration	This study
C2	*M. colombiense*	Acute lymphoblastic leukemia	Bronchoalveolar lavage + blood	This study
C3	*M. intracellulare*	Bronchiectasis	Expectoration	This study
C4	*M. intracellulare*	Neoplasia	Expectoration	This study
C5	*M. i.* subsps. *chimaera*	Pulmonary nodes and cavitary lesion	Pulmonary biopsy	This study
C6	*M. i.* subsps *chimaera*	Digestive cancer	Expectoration	This study
C7	*M. intracellulare*	Bronchiectasis	Bronchoalveolar lavage	This study
C8	*M. intracellulare*	HIV	Bronchoalveolar lavage + blood	This study
C9	*M. intracellulare*	Bronchiectasis	Bronchoalveolar lavage	This study
C10	*M. intracellulare*	Bronchiectasis	Expectoration	This study
C11	*M. intracellulare*	Bronchiectasis	Expectoration	This study
D2	*M. marseillense*	Cervical adenitis	Lymph node biopsy	This study
ATCC13950	*M. intracellulare*	Type strain		ATCC
Plasmid		Description		
pSMT3LxEGFP		Lux, GFP, (Hygromycin)		

* Chronic obstructive pulmonary disease (COPD).

**Table 2:** List of primers used in this study.

Name	Target ^1^ Gene ^1^	Sequence	Use
P1	Hsp65-F	ACCAACGATGGTGTGTCCAT	
P2	Hsp65-R	CTTGTCGAACCGCATACCCT	Subspecies
P3	rpoB-F	GGCAAGGTCACCCCGAAGGG	identification
P4	rpoB-R	AGCGGCTGCTGGGTGATCATC	
P5	Min18-F	GGATTCGGCCGCGCAATTC	
P6	Min18-R	GCCGAACCATTTGGCGAAC	
P7	Min19-F	TAGGGGCAGGTCATCGAAG	
P8	Min19-R	CATGGTTCGCCCTCTACAC	
P9	Min20-F	CGACGCCGATGACGTAAAC	
P10	Min20-R	GCTGAGCTACAGCCTCGAC	
P11	Min22-F	AGCTCGTGACGACGGAAAC	MLVA
P12	Min22-R	TCAGGAATGGGTCCGGTTC	Typing ^2^
P13	Min31-F	GCTCTATGACGACCTCAAG	
P14	Min31-R	CGACCGCATCCAGAAACAG	
P15	Min33-F	GGCGTTGAACACGTTGGTG	
P16	Min33-R	GTGCAGTTCAACCACGAAC	
P17	MIRU3 new-F	GCAAGCCGGGAACCGGATCG	
P18	MIRU3 new-R	CACCACGGTGGCCTCAAAGC	
P19	HBHA-F	TATACATATGACCATGGCGGAAAACCCGAACATCG	
P20	HBHA-intra-R	GCCGACCGCGCGGGTCTGCGA	Polymorphism analysis
P21	HBHA intra-F	TATAGAATTCCGCCAAGCTGGTGGGCATCGAGCTGCCG	and
P22	HBHA-R	CTACCTACTTCTGGGTGACCTTCTTGGC	sequencing
P23	LBP-F	ATGAATAAGGCAGAGCTC	
P24	LBP-R	CTACCGGCGGCCGCGACGCG	

^1^ Abbreviation; F: forward; R: reverse; intra: intragene. ^2^ MIN 18 through MIN 33 correspond to Tandem Repeat identified in the study of Dauchy et al. [35].

**Table 3:** Identification of HBHA and LBP by mass spectrometry analysis.

Peptides Identified by HR-MS/MS *	Start–End Position	Peptide Modification
**HBHA**		
^70^LQEDLPEQFTELR^82^	70–82	
^93^AAEGYLEAATSR^104^	93–104	
^105^YNELVER^111^	105–111	
^119^LRSQTAFEDASAR^131^	119–131	
^121^SQTAFEDASAR^131^	121–131	
^132^AEGYVDQAVELTQEALGTVASQTR^155^	132–155	
^161^AAK**me**LVGIELPGK^172^	161–172	Methylated
^164^LVGIELPGKTEATAK**me**^178^	164–178	Methylated
**LBP**		
^9^AELIDVLTQK^18^	9–18	
^25^QATAAVENVVDTIVR^39^	25–39	
^40^AVHKGDSVTITGFGVFEQR^58^	40–58	
^44^GDSVTITGFGVFEQR^58^	44–58	
^76^VKPTSVPAFRPGAQFK^91^	76–91	
^92^AVVSGAQRLPSDGPAVK^108^	92–108	
^92^AVVSGAQRLPSDGPAVK**me**R^109^	92–109	Methylated

* **me**: methylated.
